# *Plasmodium falciparum* infections and number needed to screen among refugees arriving from sub-Saharan Africa

**DOI:** 10.1093/jtm/taag017

**Published:** 2026-02-24

**Authors:** Therese Bergstrand, Katja Wyss, Isabelle Eliasson, Vilde Kaldhusdal, Ana Requena-Méndez, Anna Färnert

**Affiliations:** Department of Microbiology, Public Health Agency of Sweden, Solna, Sweden; Division of Infectious Diseases, Department of Medicine Solna, Karolinska Institutet, Stockholm, Sweden; Division of Infectious Diseases, Department of Medicine Solna, Karolinska Institutet, Stockholm, Sweden; Center for Molecular Medicine, Stockholm, Sweden; Department of Infectious Diseases, Karolinska University Hospital, Stockholm, Sweden; Division of Infectious Diseases, Department of Medicine Solna, Karolinska Institutet, Stockholm, Sweden; Center for Molecular Medicine, Stockholm, Sweden; Department of Infectious Diseases, Karolinska University Hospital, Stockholm, Sweden; Division of Infectious Diseases, Department of Medicine Solna, Karolinska Institutet, Stockholm, Sweden; Center for Molecular Medicine, Stockholm, Sweden; Department of Infectious Diseases, Karolinska University Hospital, Stockholm, Sweden; Division of Infectious Diseases, Department of Medicine Solna, Karolinska Institutet, Stockholm, Sweden; Migrant Health Research Group, Barcelona Institute for Global Health (ISGlobal, University of Barcelona), Barcelona, Spain; Division of Infectious Diseases, Department of Medicine Solna, Karolinska Institutet, Stockholm, Sweden; Center for Molecular Medicine, Stockholm, Sweden; Department of Infectious Diseases, Karolinska University Hospital, Stockholm, Sweden

**Keywords:** malaria, migrant, refugee, sub-Saharan Africa, prevalence, screening

## Abstract

**Background:**

Migrants from malaria-endemic countries, especially sub-Saharan Africa (SSA), may harbour parasites at low density in peripheral blood when relocating to non-endemic countries. This study aimed to estimate the number of subclinical malaria infections in SSA refugees arriving to Sweden, and the number needed to screen (NNS) to identify one case.

**Methods:**

Country-specific age-standardized prevalences of *Plasmodium falciparum* infections from the Malaria Atlas Project (MAP) were applied to demographic data on SSA refugees who had recently arrived in Sweden, 2015–22. Indirect estimates of the number of asymptomatic *P. falciparum* infections and NNS in different age groups were stratified by country transmission levels.

**Results:**

In total, 3930 (7%) of the 56 248 asylum applicants or quota refugees arriving from SSA during 2015–22 were estimated to be infected with *P. falciparum* parasites, including 1789 children and 2141 adults, of which 851 women of reproductive age. The estimated total number of individuals with asymptomatic *P. falciparum* infections arriving in Sweden per year was 491 (range 356–546), and 80% of infections were in individuals from high and moderate-transmission countries. The NNS was estimated to 2–14 in individuals arriving from countries with high and moderate transmission levels.

**Conclusions:**

Modelled estimates of *P. falciparum* rates in newly arrived refugees correspond to previously reported screening parasite prevalence in SSA migrant populations. A low number of migrants need to be screened to detect one case in populations arriving from high-moderate transmission areas. These estimates can guide screening strategies and could reduce the burden of malaria in migrants from SSA.

## Introduction

Malaria causes high morbidity and mortality, especially in sub-Saharan Africa (SSA), holding 94% of cases and 95% of deaths.^1^ In addition, asymptomatic infections with malaria parasites are prevalent as a result of partial immunity after repeated exposure.^2^ These asymptomatic infections, typically characterized by low parasite density and absence of fever, can nonetheless lead to significant health issues, including anaemia, splenomegaly and severe complications during pregnancy.^3–8^ Additionally, such infections may progress to febrile malaria and have long-term health consequences, such as lymphoma and cognitive impairment in children.^8–10^ Migrants from endemic areas may harbour blood-stage malaria parasites as they arrive in non-endemic areas and asymptomatic infections may persist after arrival.^11^ If febrile malaria develops, it is generally suspected, diagnosed and treated, at least during the first months after arrival.^5^ Otherwise, the infection is likely to be unrecognized, and the diagnosis may be missed or delayed, leading to potential complications.^12^^,^^13^ There is also risk of transmission through blood products, transplants or vertical transmission during pregnancy, as well as, although very limited, by competent *Anopheles* species under optimal conditions.^14–18^

Screening for malaria in migrants is generally not implemented in non-endemic countries, except for Australia,^19^ whilst the US Centre for Disease Control (CDC) recommends presumptive treatment.^20^ A recent study of 789 SSA migrants screened in Sweden showed that 10% of those attending a voluntary migrant health assessment tested positive for *Plasmodium spp* by polymerase chain reaction (PCR), with especially high prevalence, 22% in adults and 36% in children arriving from Uganda.^21^ In addition, a systematic review and meta-analysis of screening studies reported a pooled PCR-prevalence of 8% in migrants from SSA.^22^

Estimates of the number of potentially infected migrants arriving to non-endemic areas as well as number needed to screen (NNS), i.e. how many migrants that would have to be screened to find one positive case, are needed to inform future public health strategies. The aim of this study was to estimate malaria parasite prevalence, NNS and number of asymptomatically infected migrants from SSA arriving in Sweden.

## Methods

### Study design and study population

This was a descriptive cross-sectional study using demographic migrant data in Sweden 2015–22, together with annual modelled country-specific prevalences for *P. falciparum*.

Targeted migrants included asylum-seekers and quota refugees with citizenship in any of the 52 malaria-endemic countries in Africa (according to the United Nations Statistics Division, and including Sudan).^23^ Other migrant categories and family members of asylum-seekers who received residence permits were not included due to unavailable data (e.g. date of arrival in Sweden).

The study was performed following the STROBE guidelines (Supplementary Table 1). No ethical approval was needed since only aggregated data and no personal data were handled.

### Data sources

Aggregated demographic data, including year of arrival, country of citizenship, sex and age in 5-year intervals, were provided by the Statistics Service Unit at the Swedish Migration Agency.^24^

**Figure 1:**
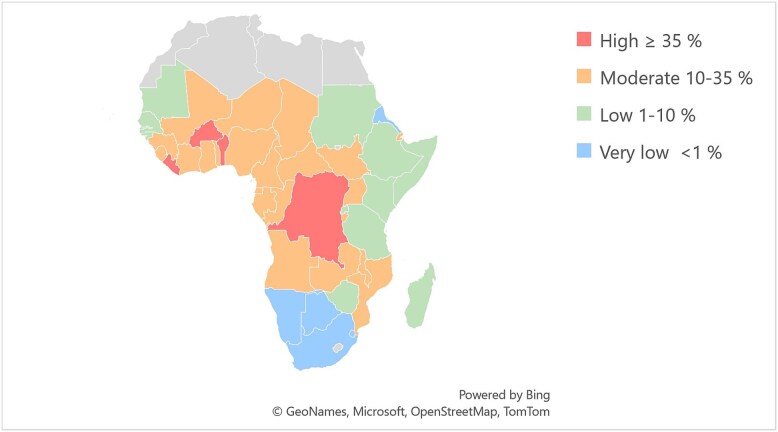
Countries in SSA per transmission level as defined by the World Health Organization^10^ based on the respective countries parasite prevalence rate for *P. falciparum* malaria in children 2–10 years of age in 2020 from the MAP.^25^

Country-specific modelled age-standardized *P. falciparum* prevalence estimates in the countries of origin were provided by the Malaria Atlas Project (MAP).^25^ The modelled estimates from MAP are based on observed parasitaemia in cross-sectional survey data such as Demographic and Health Surveys (DHS), including Malaria Indicator Surveys (MIS).^26^ Parasite rates were available for *P. falciparum* in children 2–10 years of age (PfPR_2–10_) and standardized to microscopy, if the method of detection was rapid diagnostic test (RDT).^27^ For adults, the rates (PfPR_16–80_) were calculated as described below. Modelled prevalence in each country of origin per year of arrival was applied for the respective years 2015–20. For 2021 and 2022, the latest updated parasite rates of 2020 from MAP were used. Parasite rates per country and year are listed in Supplementary Table 3. Countries of origin were stratified by transmission level based on the modelled PfPR_2–10_ for 2020; to high (≥35%), moderate (10–35%), low (1–10%) and very low (<1%) levels according to WHO^10^ (Figure 1, Supplementary Table 2). During the study period for which MAP prevalences were available, the country-specific prevalences for a few countries changed between the levels, for instance, the Democratic Republic of the Congo (DRC) changed from moderate to high, however most countries’ transmission remained in the same level (Supplementary Table 3).

### Data analyses

#### Number of estimated asymptomatic infections of P. falciparum

A model to estimate parasite prevalence in migrants based on demographic data, including countries of origin, was performed as previously described for Chagas disease.^28^ The estimated number of individuals arriving to Sweden with asymptomatic *P. falciparum* infections was calculated by multiplying the number of migrants arriving during the study period from that country by the corresponding annual country-specific prevalence from MAP. For children (0–15 years), *P. falciparum* parasite rates for 2–10 years (PfPR_2–10_) were used. For adults, prevalences were calculated for the age group 16–80 years (PfPR_16–80_) using the MAP:s R package malariaAtlas version 1.5.1 with provided sample-weights,^29^ and the specific function convertPrevalence re-map PfPR from one age group to another. This algorithm standardizes prevalence estimates across ages based on the relationship between age and parasite rate (PfPR), where the probability to detect an active infection changes with age and immunity. The parasite rate peaks around age 2 years, where it remains until age 10 before declining, hence providing basis for PfPR_2–10_.^30^ The algorithm compensates for loss of sensitivity by microscopy in older ages, where immunity limits the parasitaemia often to sub-microscopic levels. To ensure that estimates are representative of the population structure for different countries, the R package includes country-specific sample weights. For children, the estimate PfPR_2–10_ are based on observational data expressed with 95% CI reflecting the uncertainty in these estimates. From this 95% CI, minimum and maximum values were derived for the estimates. For adults, the calculated prevalence PfPR_16–80_ are standardized point estimates that provide bias-adjusted comparisons across age groups and countries, hence no 95% CI and corresponding minimum and maximum values.

### Estimated NNS

To estimate NNS depending on transmission level in country of origin, a simple model was used based on prevalence described by WHO in the ‘Operational Handbook on the WHO TB Knowledge sharing platform’.^31^ The NNS is the inverse of the prevalence in the specific risk group and was here calculated in adults and children based on parasite prevalence data in 2020 (the latest available prevalence data in MAP).

Data analyses were performed using STATA/SE 18.0 (StataCorp LLC, USA) and R 4.3.3.

## Results

### Estimated number of migrants with asymptomatic *P. falciparum*

**Figure 2:**
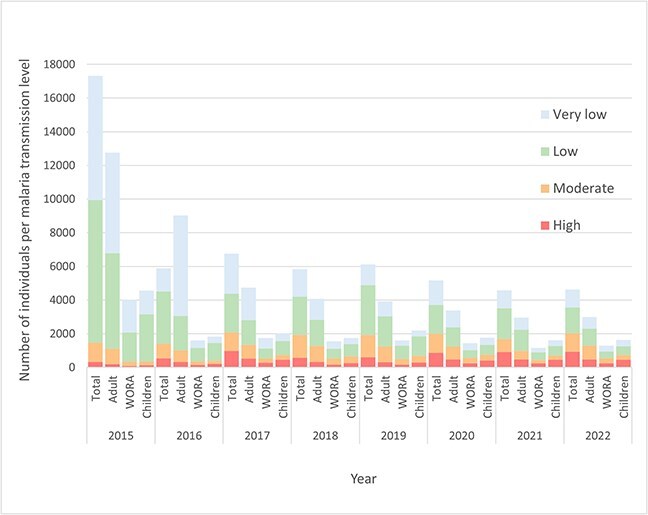
Number of asylum-seekers and quota refugees arriving in Sweden per malaria transmission level per year in total (all ages), for adults (≥ 16 years of age), women of reproductive age (WORA) (16–50 years of age) and children (>16 years of age). Transmission level high (≥35%), moderate (10–35%), low (1–10%) and very low (<1%) according to World Health Organization^10^ based on parasite prevalence rate for *P. falciparum* in children two to ten years of age in 2020 from the MAP^25^ per respective country.

During the years 2015–22, 302 083 individuals applied for asylum or arrived as quota refugees in Sweden, of which 56 248 originated from malaria-endemic countries in SSA and were included in the model (Table 1). In addition, 24 421 family-members of asylum-seekers from SSA received a residence permit but could not be included due to unavailable data. Among the 56 248 migrants, >17 000 arrived in 2015, whereas between 4500 and 6700 individuals arrived annually during 2016–22 (Figure 2 and Supplementary Table 2).

**Table 1:** Estimated number of newly arrived asylum seekers and quota refugees (migrants) with asymptomatic *P. falciparum* infection (based on microscopy) and NNS to detect one infected individual, per malaria transmission level.

**Malaria transmission** **level** ^**a**^	**Number of migrants** **arriving 2015–20** ***n* (%)**				***P. falciparum*** **prevalences countries of origin 2020 MAP % (min-max)**		**Estimated number of *P. falciparum*** **infected migrants arriving 2015–2020** ^**c**^ ***n* (%)**				**Numbers needed to screen** ^**d**^ ***n* range**	
	Total^e^	Adults ^f^	WORA^g^	Children^h^	PfPR_2–10_^i^	PfPR_16-80_^j^	Total^e^	Adults^f^	WORA^g^	Children^h^	Adults	Children
**High** ≥35%	5708 (10.1)	3069 (7.9)	1522 (10.6)	2639 (15.2)	35.5–40.1	23.6–26.6	1568 (39.9)	683 (31.9)	338 (39.7)	885 (49.5)	4	2–3
**Moderate** 10–35%	8776 (15.6)	6412 (15.5)	2283 (15.9)	2364 (13.6)	10.5–33.4	7.0–22.2	1606 (40.9)	1017 (46.5)	359 (42.2)	589 (32.9)	5–14	3–9
**Low** 1–10%	24 241 (43.1)	15 972 (41.1)	5805 (40.4)	8269 (47.7)	1.9–7.9	1.3–5.3	679 (17.3)	390 (18.2)	136 (16.0)	289 (16.2)	19–78	13–52
**Very low** < 1%	17 523 (31.2)	13 447 (34.6)	4756 (36.3)	4076 (23.5)	0.01–0.9	0.01–0.60	77 (2.0)	51 (2.4)	19 (2.2)	26 (1.5)	167–11 912	111–7905
**Total**	56 248	38 900	14 366	17 348	-	-	3930	2141	851	1789	4–11 912	2–7905

^a^Transmission level high (≥35%), moderate (10–35%), low (1–10%) and very low (<1%) according to the World Health Organization (10) based on parasite prevalence rate for *P. falciparum* malaria in children 2–10 years of age in 2020 (PfPR_2–10_ 2020) from the MAP (25) per respective country.

^b^Parasite prevalence rate for *P. falciparum* in children 2–10 years of age in 2020 (PfPR_2–10_ 2020) and adults (PfRP_16–80_ 2020) respectively from the MAP.

^c^Estimated number of asymptomatic infections of *P. falciparum* per country per year from prevalence per year 2015, 2016, 2017, 2018, 2019 and 2020. For 2021 and 2022 prevalence for 2020 was used.

^d^NNS based on the range of minimum and maximum prevalence per transmission level from the MAP prevalence for 2020 for children PfPR_2–10_ and adults PfRP_16–80_ respectively.

^e^Includes all ages.

^f^≥16 years of age.

^g^Women of reproductive age (WORA) 16–50 years of age.

^h^<16 years of age.

^i^Range between minimum and maximum parasite prevalence rate for *P. falciparum* malaria in children 2–10 years of age in 2020 (PfPR_2–10_ 2020) from the MAP per level (for 95% CI, see Supplementary Table 3).

^j^Range between minimum and maximum prevalence for adults in 2020 (PfPR_16–80)_ from the MAP per level

Overall, 5708 (10.1%) migrants came from countries with high malaria transmission, 8776 (15.6%) from moderate, 24 241 (43.1%) from low and 17 523 (31.2%) from countries with very low transmission levels as defined in 2020 (Table 1).

**Figure 3:**
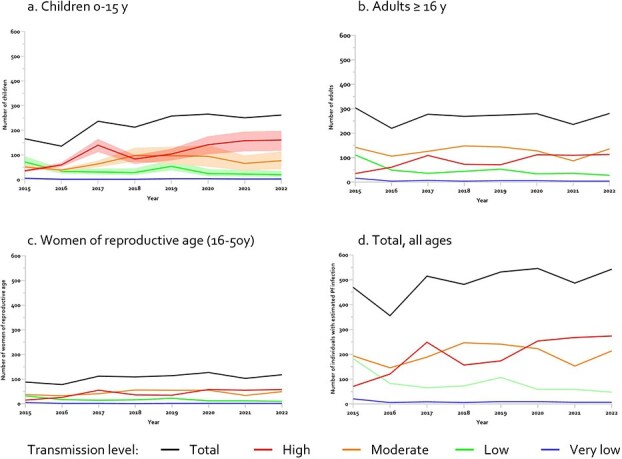
Estimated number of individuals with asymptomatic *P. falciparum* infection arriving in Sweden per year per malaria transmission level high (≥35%), moderate (10–35%), low (1–10%) and very low (<1%) of the countries of origin for a. Children b. Adults. c. Women of reproductive age, and d. Total, all ages. The estimates and ranges (minimum and maximum values in shaded area) for children were derived from the *P. falciparum* parasite prevalence in children 2–10 years (PfPR_2–10_) provided by the MAP and included 95% CI, whereas 95% CI were not available for adults.

Based on the country-specific parasite prevalences for respective years, a mean of 491 (range 356–546) individuals, including all ages, were potentially infected with *P. falciparum* per year (Figure 3), resulting in total 3930 infected individuals during 2015–22, corresponding to an overall parasite prevalence of 7% in the whole population (*n* = 56 248). Excluding 2015, with exceptionally high arrival rates, the mean was 494 and the total 3460 infected individuals, resulting in a prevalence of 8.9% (*n* = 38 931) 2016–22.

Model estimates of the annual mean number of children (0–15 y) with potential *P. falciparum* was 224 (range 136–266) per year, resulting in a total 1789 over the whole period, corresponding to a prevalence of 10.3% in all children (Figure 3, Table 1).

In adults, the estimated mean number was 268 (range 220–304) per year, with a total of 2141 infected during the study period, corresponding to a prevalence of 5.5% (Figure 3, Table 1). For women of reproductive age, a mean of 106 (range 78–127) infected women arrived per year, with a total of 851 (5.9%) for 2015–22 (Figure 3, Table 1). Citizenship in countries with high and moderate transmission level accounted for around 80% of estimated cases in children (n = 1474, 82.4%), adults (*n* = 1700, 79.4%), as well as women of reproductive age (*n* = 697, 81.9%) (Figure 3).

### Estimated numbers needed to screen

The estimated NNS to detect one case in children from high transmission countries was 2–3, and 3–9 for moderate, 13–52 for low and 111–7905 for very low (Table 1). For adults, the NNS was 4 for high transmission countries, 5–14 for moderate, 19–78 for low and 167–11 912 for very low transmission countries (Table 1).

## Discussion

Using demographic data on asylum seekers and refugees arriving to Sweden together with malaria parasite prevalence data in countries of origin, we estimated that on average 490 migrants arriving from SSA to Sweden per year may have been harbouring *P. falciparum* parasites detectable by microscopy during the years 2015–22. We found that 80% of these estimated asymptomatic *P. falciparum* infections were in individuals coming from countries with high and moderate malaria transmission, and the NNS to identify one infection in these groups was only 2–9 in children and 4–14 in adults.

The estimated parasite prevalence of 7% in newly arrived migrant population in Sweden during this period corresponds well with findings in studies where malaria screening was performed.^21^^,^^22^ A pooled prevalence of 8.3% by PCR and 3.3% by microscopy was found in a meta-analysis of 14 studies that screened migrants from SSA in non-endemic countries.^22^ Screening by PCR in Sweden also resulted in 10% malaria parasite prevalence in newly arrived migrants.^21^

The number of migrants from SSA arriving in Sweden varied during the study period, both in numbers and countries of citizenship. However, the estimated number of asymptomatic *P. falciparum* infections was rather similar from year to year. During a period of increased migration in 2015, many arrived from SSA countries, mostly from low transmission countries (Eritrea, Ethiopia and Somalia), yet the estimated number of *P. falciparum* infections was similar to the later years when fewer came but from countries with higher transmission (e.g. DRC). Nonetheless, these estimates are restricted to *P. falciparum*, yet other species contribute to the burden of malaria in migrants. Although *P. falciparum* was most prevalent, *Plasmodium ovale* spp and *P. malariae* were also found alone or as mixed infections in migrants tested in Sweden.^21^  *Plasmodium* species may also vary with time, migrant routes and populations, as demonstrated by the high incidence of clinical cases of *Plasmodium vivax* reported in Eritrean migrants arriving in Europe 2014–15.^32–34^

This is, to our knowledge, the first study estimating asymptomatic malaria parasite prevalence in refugee populations. The modelled estimates of infected individuals could be both overestimates and underestimates. Previous treatment or other interventions during migration could lead to reduced parasite prevalence on arrival. However, high malaria transmission levels in refugee camps compared to local populations,^35^ could also lead to higher infection rates. Moreover, family members of asylum-seekers were not included due to missing data. Extrapolating the overall parasite prevalence of 7% on the 24 441 family members arriving 2015–22 would result in an additional total 1710 infected individuals, and 244 per year.

Children and pregnant women are the most vulnerable to subclinical malaria infections. During the study period, 220 children with asymptomatic infections were estimated to have arrived in Sweden annually. The findings estimate an overall prevalence in children of 10.3% by microscopy, similar to the 12.1% (all species) with PCR in children screened at the migrant health assessment units in the Swedish screening study^21^ but higher than a study in Germany that reported a prevalence of 2.3% (all species) in refugee children from SSA screened by RDT after arrival.^36^ Countries of origin are important, and in children from Uganda, the modelled PfPR_2–10_ by MAP for 2020 was 23%, which is comparable to the 35% (all species) PCR positivity in children arriving from Uganda in our previous migrant screening study.^21^

For women of reproductive age, around 100 women arriving from SSA to Sweden per year were estimated to have asymptomatic *P. falciparum* infections, with potentially severe consequences for both the woman and the child in case of pregnancy.^8^ Newly arrived pregnant women would neither be tested in migrant health or antenatal clinics, nor receive intermittent preventive treatment (IPTp) in Sweden, which is recommended by WHO in endemic areas.^10^ Additionally, malaria-related complications in pregnancies up to 5 years after arrival have been reported in a systematic review.^14^

The NNS is a crude estimate based on the prevalence of an infection in countries of origin that can guide screening programme design.^31^ For latent tuberculosis infection (LTBI) screening of migrants in Sweden is done on individuals from high-incidence countries (TB incidence ≥100 per 100 000), such as in SSA, and the LTBI prevalence has been found to be 25%,^37^ thus 4 people needed to be tested to find one case. This is similar to estimates in our study, with 2–3 children and 4 adults from a high malaria transmission setting needed to screen to find one *P. falciparum* infection.

Our estimates suggest that ~500 newly arrived migrants per year are infected with *P. falciparum*, yet only a fraction of these infections is clinically detected. According to national surveillance data from the Public Health Agency of Sweden, the number of reported malaria cases during the same period ranged from 138 to 249 annually, with an average of 200 cases.^38^ A retrospective review of clinical records indicates that ~30% of these malaria cases occurred in individuals who had lived in Sweden for <1 year, corresponding to ~40–75 cases annually in newly arrived migrants (unpublished). This suggests that only 10% of the estimated *Plasmodium* infections are diagnosed and reported, and most infections remain undetected.

Implications of these findings are that the estimate of a high number of asymptomatic *P. falciparum* infections indicates a hidden public health burden in non-endemic countries that should be addressed. The most important reason to detect these infections is to avoid the possible consequences to the individual. Although most infections are asymptomatic and subclinical, fever and other health problems and pregnancy complications^14^ may occur, and anaemia and splenomegaly are common.^39^ There is also a risk of transfer through blood transfusion or organ transplant.^16^^,^^17^ Moreover, although reintroduction of malaria is highly unlikely, sporadic transmission could occur under optimal conditions, where competent vectors are present, such as in southern Europe.^40^  *Anopheles* species, e.g. *An. plumbeus* a potential vector for both *P. vivax* and *P. falciparum,* has been found even in countries in northern Europe, including Sweden.^18^

This study has several limitations. First, the estimates are only at country level and malaria transmission can vary considerably within a country. Moreover, the prevalences were based on country of citizenship, although many migrants may have transited through or resided in other countries over long periods before arrival. Further limitations concern the use of MAP prevalences based on microscopy and converted RDT results, which only detect ~50% of PCR-positive cases.^3^^,^^4^ Hence, using PCR-based estimates, the number of asymptomatic infections may have been twice as high. Moreover, the study was restricted to *P. falciparum,* the only species for which data was available through MAP for the countries of interest in this study. However, other *Plasmodium* species are prevalent in endemic areas and also detected in screened migrants, as discussed above.^21^ The latest year available for parasite prevalence estimates through MAP was 2020.^25^ Moreover, MAP data for adults were based on calculated prevalence and did not include 95% CI, as was available for children. Finally, only part of the migrant population from SSA was included and not family members of asylum-seekers or other migrants relocating for labour or education.

The strength of this study was that detailed demographic data were provided for migrant populations in age intervals, sex and per country per year, allowing for estimates overall as well as for children and women of reproductive age separately. In addition, the use of prevalence per year for each of the countries allowed to account for changes in prevalence as well as migration patterns, and number of migrants arriving from areas of different levels of exposure.

The number of migrants was estimated based on newly arrived first-time applicants for asylum and quota refugees, hence not including relatives or other migrant groups. Although the duration of asymptomatic *P. falciparum* infections is not established, case reports of malaria several years after last exposure suggest that infections can last a long time.^11^ Hence, the total number of individuals carrying malaria parasites in Sweden may be even higher. To what extent infections are cleared without treatment and the potential clinical consequences of asymptomatic infections need to be established.

Further studies addressing the impact of asymptomatic infections in migrants, as well as estimates on feasibility and cost-effectiveness of screening interventions, are needed. Moreover, the clinical and public health consequences of these infections are yet to be further understood, including the duration of asymptomatic infections. Conversely, based on WHO guidelines in endemic areas, individuals with asymptomatic infections should be offered treatment at least in risk groups such as children and pregnant women.^10^

## Conclusions

Modelled estimates of asymptomatic *P. falciparum* infections in populations newly arrived in Sweden from SSA contribute to the increasing documentation about subclinical malaria infections in migrant populations. The low NNS in high and moderate-transmission countries suggests that a screening intervention could be effective in finding asymptomatic infections in high-risk groups.

## Supplementary Material

Bergstrand_Supplementary_material_251202_AF_revised_proof_taag017

## Data Availability

The data from the Malaria Atlas Project and the aggregated migrant statistics data underlying this article are available as online supplementary material (Supplementary Tables 2 and 3). The migrant population statistic data per country will be shared on reasonable request to the corresponding author.
