# Supplementary material for: Plasmodium falciparum infections and number needed to screen among refugees arriving from sub-Saharan Africa
Source: J Travel Med. 2026 Feb 24;33(3):taag017. doi: 10.1093/jtm/taag017 (PMC13042227; doi:10.1093/jtm/taag017)
Supplement: Bergstrand_Supplementary_material_251202_AF_revised_proof_taag017 [file bergstrand_supplementary_material_251202_af_revised_proof_taag017.docx]

**Supplementary material**

**Supplementary Table 1. STROBE checklist for observational studies**

|  | | Item No |  | Recommendation | |  | |
| --- | --- | --- | --- | --- | --- | --- | --- |
| **Title and abstract** | | 1 |  | (*a*) Indicate the study’s design with a commonly used term in the title or the abstract | | Yes, Page 4 | |
|  |  |  |  | (*b*) Provide in the abstract an informative and balanced summary of what was done and what was found | | Yes, Page 2 | |
|  | Introduction | | | | |  | |
| Background/rationale | | 2 |  | Explain the scientific background and rationale for the investigation being reported | | Yes, Page 3-4 | |
| Objectives | | 3 |  | State specific objectives. including any prespecified hypotheses | | Yes, Page 4 | |
|  | Methods | | | | |  | |
| Study design | | 4 |  | Present key elements of study design early in the paper | | Yes, Page 4 | |
| Setting | | 5 |  | Describe the setting. locations. and relevant dates. including periods of recruitment. exposure. follow-up. and data collection | | Yes, Page 4-5 | |
| Participants | | 6 |  | (*a*) Give the eligibility criteria. and the sources and methods of selection of participants | | Yes, Page 4-5 | |
| Variables | | 7 |  | Clearly define all outcomes. exposures. predictors. potential confounders. and effect modifiers. Give diagnostic criteria. if applicable | | Yes, Page 5-6 | |
| Data sources/ measurement | | 8* |  | For each variable of interest. give sources of data and details of methods of assessment (measurement). Describe comparability of assessment methods if there is more than one group | | Yes, Page 4-5 | |
| Bias | | 9 |  | Describe any efforts to address potential sources of bias | | Yes, Page 7 | |
| Study size | | 10 |  | Explain how the study size was arrived at | | NA^1^ | |
| Quantitative variables | | 11 |  | Explain how quantitative variables were handled in the analyses. If applicable. describe which groupings were chosen and why | | Yes, Page 5-6 | |
| Statistical methods | | 12 |  | (*a*) Describe all statistical methods. including those used to control for confounding | | Yes, Page 5-6 | |
|  |  |  |  | (*b*) Describe any methods used to examine subgroups and interactions | | Yes, Page 5-6 | |
|  |  |  |  | (*c*) Explain how missing data were addressed | | NA^2^ | |
|  |  |  |  | (*d*) If applicable. describe analytical methods taking account of sampling strategy | | - | |
|  |  |  |  | (*e*) Describe any sensitivity analyses | | NA^1^ | |
|  | **Supplementary Table 1, cont.**  Results | | | |  | |  |
| Participants | | 13* |  | (a) Report numbers of individuals at each stage of study—eg numbers potentially eligible. examined for eligibility. confirmed eligible. included in the study. completing follow-up. and analysed | | Yes, Page 6-7 | |
|  |  |  |  | (b) Give reasons for non-participation at each stage | | NA^1^ | |
|  |  |  |  | (c) Consider use of a flow diagram | | NA | |
| Descriptive data | | 14* |  | (a) Give characteristics of study participants (eg demographic. clinical. social) and information on exposures and potential confounders | | Yes, Table 1, Figure 2 | |
|  |  |  |  | (b) Indicate number of participants with missing data for each variable of interest | | NA^2^ | |
| Outcome data | | 15* |  | Report numbers of outcome events or summary measures | | Yes, Table 1, Figure 3 | |
| Main results | | 16 |  | (*a*) Give unadjusted estimates and. if applicable. confounder-adjusted estimates and their precision (eg. 95% confidence interval). Make clear which confounders were adjusted for and why they were included | | Yes, Table 1 | |
|  |  |  |  | (*b*) Report category boundaries when continuous variables were categorized | | NA^3^ | |
|  |  |  |  | (*c*) If relevant. consider translating estimates of relative risk into absolute risk for a meaningful time period | | NA^1^ | |
| Other analyses | | 17 |  | Report other analyses done—eg analyses of subgroups and interactions. and sensitivity analyses | | NA^1^ | |
|  | Discussion | | | | |  | |
| Key results | | 18 |  | Summarise key results with reference to study objectives | | Yes, Page 8-9 | |
| Limitations | | 19 |  | Discuss limitations of the study. taking into account sources of potential bias or imprecision. Discuss both direction and magnitude of any potential bias | | Yes, Page 11 | |
| Interpretation | | 20 |  | Give a cautious overall interpretation of results considering objectives. limitations. multiplicity of analyses. results from similar studies. and other relevant evidence | | Yes, Page 8-12 | |
| Generalisability | | 21 |  | Discuss the generalisability (external validity) of the study results | | Yes, Page 12 | |
|  | Other information | | | | |  | |
| Funding | | 22 |  | Give the source of funding and the role of the funders for the present study and. if applicable. for the original study on which the present article is based | | NA^4^ | |

^1^ Not applicable since this a descriptive study. ^2^ No missing data in the dataset. ^3^ Only categorical variables in the dataset.

**Supplementary Table 2**. Description of the study population overall and per transmission level

| **Number of asylum seekers and quota refugees from Sub-Saharan Africa** **arriving to Sweden** **2015-2022, n (%)** | | | | | | | | | |
| --- | --- | --- | --- | --- | --- | --- | --- | --- | --- |
| **Year** | **2015-2022**  N=56248 | **2015**  n=17317 | **2016**  n=5878 | **2017**  n=6754 | **2018**  n=5830 | **2019**  n=6114 | **2020**  n=5158 | **2021**  n=4574 | **2022**  n=4623 |
| **Age category** |  |  |  |  |  |  |  |  |  |
| < 16 years | 17 348  (30.8) | 4 562 (26.3) | 1 820 (26.9) | 2 008 (29.7) | 1 754  (30.1) | 2 187  (35.8) | 1 769 (34.3) | 1 616  (35.3) | 1 632  (35.3) |
| ≥ 16 years | 38 900  (69.2) | 12 755 (73.7) | 4 058 (69.0) | 4 746 (70.3) | 4 076  (69.9) | 3 927  (64.2) | 3 389 (65.7) | 2958  (64.7) | 2991 (64.7) |
| Women repr age^a^ | 14 366  (25.5) | 4 015 (23.2) | 1 601 (27.2) | 1 741 (25.8) | 1542  (26.4) | 1593  (26.1) | 1432 (27.8) | 1161  (25.4) | 1281 (42.8) |
| **Sex** |  |  |  |  |  |  |  |  |  |
| Female | 23 423  (41.6) | 5 854 (33.8) | 2 647 (45.0) | 2 912 (43.1) | 2 563  (44.0) | 2 807  (45.9) | 2 390 (46.3) | 2 083  (45.5) | 2 167 (46.9) |
| Male | 32 825  (58.4) | 11463 (66.2) | 3231 (55.0) | 3842 (56.9) | 3267  (56.0) | 3307  (54.1) | 2768 (53.7) | 2491  (54.5) | 2456 (53.1) |
| **Transmission**^b^ |  |  |  |  |  |  |  |  |  |
| **High all** | 5 708  (10.1) | 321  (1.9) | 529  (9.0) | 977  (14.4) | 573  (9.8) | 597  (9.8) | 870  (16.9) | 910  (19.9) | 931 (20.1) |
| < 16 years | 2 639  (46.2) | 131  (40.8) | 212 (40.1) | 453  (46.4) | 250  (43.6) | 295  (49.4) | 399  (45.9) | 446  (49.0) | 453 (48.7) |
| ≥ 16 years | 3 069  (53.8) | 190  (59.2) | 317 (59.9) | 524  (53.6) | 323  (56.4) | 302  (50.6) | 471  (54.1) | 464  (51.0) | 478 (51.3) |
| Women repr age^a^ | 1 522  (26.7) | 83  (25.9) | 136 (25.7) | 268  (37.4) | 161  (28.1) | 151  (25.3) | 245  (28.2) | 234  (25.7) | 244 (26.2) |
| **Moderate all** | 8 776  (15.6) | 1 151 (6.6) | 874 (14.9) | 1 094 (16.2) | 1 352  (23.2) | 1 326  (21.7) | 1 119 (21.7) | 772  (16.9) | 1 088 (23.5) |
| < 16 years | 2 364  (26.9) | 232  (20.2) | 180 (20.6) | 275  (25.1) | 406  (30.0) | 381  (28.7) | 351  (31.4) | 254  (32.9) | 285 (26.2) |
| ≥ 16 years | 6 412  (73.1) | 919  (79.8) | 694 (79.4) | 819  (74.9) | 946  (70.0) | 945  (71.3) | 768  (68.6) | 518  (67.1) | 803 (73.8) |
| Women repr age^a^ | 2 283  (26.0) | 254  (22.1) | 227 (26.0) | 270  (24.7) | 362  (26.8) | 352  (26.5) | 325  (29.0) | 204  (26.4) | 289 (26.6) |
| **Low all** | 24 241  (43.1) | 8 476 (48.9) | 3 115 (53.0) | 2 308 (34.2) | 2 280  (39.1) | 2 963  (48.5) | 1 726 (33.5) | 1 829  (40.0) | 1 544 (33.4) |
| < 16 years | 8 269  (34.1) | 2794 (33.0) | 1066 (34.2) | 845  (36.6) | 726  (31.8) | 1165  (39.3) | 581  (33.7) | 572  (31.3) | 520 (33.7) |
| ≥ 16 years | 15 972  (65.9) | 5682 (67.0) | 2049 (65.8) | 1463 (63.4) | 1554  (68.2) | 1798  (60.7) | 1145 (66.3) | 1257  (68.7) | 1024 (66.3) |
| Women repr age^a^ | 5 805  (23.9) | 1739 (20.5) | 800 (25.7) | 580  (25.1) | 589  (25.8) | 777  (26.2) | 453  (26.2) | 461  (25.2) | 406 (26.3) |
| **Very low all** | 17 523  (31.2) | 7369 (42.6) | 1360 (23.1) | 2375 (35.2) | 1625  (27.9) | 1228  (20.1) | 1443 (28.0) | 1 063  (23.2) | 1 060 (22.9) |
| < 16 years | 4 076  (23.3) | 1405 (19.1) | 362 (26.6) | 435  (18.3) | 372  (22.9) | 346  (28.2) | 438  (30.4) | 344  (32.4) | 374 (35.3) |
| ≥ 16 years | 13 447  (76.7) | 5 964 (80.9) | 998 (73.4) | 1 940  (81.7) | 1 253  (77.1) | 882  (71.8) | 1 005 (69.6) | 719  (67.6) | 686 (64.7) |
| Women repr age^a^ | 4756  (27.1) | 1939 (26.3) | 438  (32.2) | 623  (26.2) | 430  (26.5) | 313  (25.5) | 409  (28.3) | 262  (24.6) | 342 (32.3) |

^a^ Subgroup of adults, women of reproductive age (16-50 years) in absolute numbers and proportion of all per year or level

^b^ Population stratified by country prevalence into malaria transmission level high, moderate, low, very low according to World Health Organization(2) based on parasite prevalence rate for *Plasmodium falciparum* malaria in children two to ten years of age in 2020 from the Malaria Atlas Project (31) per respective country.

**Supplementary Table 3.** *Plasmodium falciparum* parasite rate in children 2-10 years of age (PfPR_2-10_ (95% CI)) and adults 16-80 years of age (PfPR_16-80_) in countries of origine according to transmission level and year based on data from the Malaria Atlas Project.

|  |  | **2015** | | **2016** | | **2017** | | **2018** | | **2019** | | **2020** | |
| --- | --- | --- | --- | --- | --- | --- | --- | --- | --- | --- | --- | --- | --- |
|  | **Country** | **PfPR 2-10y** | **PfPR 16-80y** | **PfPR 2-10y** | **PfPR 16-80y** | **PfPR 2-10y** | **PfPR 16-80y** | **PfPR 2-10y** | **PfPR 16-80y** | **PfPR 2-10y** | **PfPR 16-80y** | **PfPR 2-10y** | **PfPR 16-80y** |
| **High** | Benin | 0.35 (0.30-0.40) | 0.23 | 0.39 (0.35-0.43) | 0.26 | 0.39 (0.36-0.41) | 0.26 | 0.38 (0.35-0.40) | 0.25 | 0.37 (0.32-0.42) | 0.24 | 0.36 (0.26-0.46) | 0.24 |
|  | Burkina Faso | 0.38 (0.34-0.44) | 0.25 | 0.33 (0.25-0.42) | 0.22 | 0.33 (0.21-0.45) | 0.22 | 0.33 (0.20-0.48) | 0.22 | 0.32 (0.19-0.47) | 0.21 | 0.35 (0.17-0.53) | 0.24 |
|  | DRC | 0.27 (0.25-0.29) | 0.18 | 0.28 (0.24-0.32) | 0.19 | 0.31 (0.25-0.36) | 0.21 | 0.34 (0.26-0.40) | 0.22 | 0.35 (0.26-0.42) | 0.23 | 0.36 (0.26-0.44) | 0.24 |
|  | Liberia | 0.35 (0.28-0.43) | 0.23 | 0.39 (0.35-0.43) | 0.26 | 0.44 (0.35-0.54) | 0.29 | 0.44 (0.30-0.57) | 0.29 | 0.42 (0.25-0.60) | 0.28 | 0.40 (0.20-0.64) | 0.27 |
| **Moderrate** | Angola | 0.12 (0.12-0.14) | 0.08 | 0.14 (0.13-0.15) | 0.09 | 0.17 (0.15-0.20) | 0.11 | 0.19 (0.15-0.25) | 0.13 | 0.20 (0.14-0.28) | 0.14 | 0.23 (0.15-0.35) | 0.16 |
|  | Burundi | 0.15 (0.13-0.17) | 0.10 | 0.18 (0.17-0.20) | 0.12 | 0.21 (0.19-0.22) | 0.14 | 0.26 (0.22-0.29) | 0.17 | 0.27 (0.22-0.33) | 0.18 | 0.29 (0.21-0.39) | 0.20 |
|  | Cameroon | 0.22 (0.16-0.27) | 0.15 | 0.21 (0.18-0.25) | 0.14 | 0.20 (0.18-0.23) | 0.14 | 0.20 (0.19-0.21) | 0.13 | 0.20 (0.17-0.23) | 0.13 | 0.22 (0.14-0.31) | 0.15 |
|  | CAR | 0.36 (0.19-0.67) | 0.24 | 0.34 (0.17-0.64) | 0.23 | 0.34 (0.14-0.65) | 0.23 | 0.34 (0.13-0.65) | 0.23 | 0.34 (0.13-0.67) | 0.22 | 0.33 (0.13-0.63) | 0.22 |
|  | Chad | 0.13 (0.07-0.21) | 0.09 | 0.13 (0.07-0.22) | 0.09 | 0.13 (0.08-0.25) | 0.09 | 0.14 (0.09-0.26) | 0.09 | 0.14 (0.08-0.24) | 0.09 | 0.14 (0.07-0.22) | 0.09 |
|  | Congo | 0.15 (0.10-0.21) | 0.10 | 0.16 (0.09-0.25) | 0.11 | 0.19 (0.09-0.31) | 0.13 | 0.21 (0.11-0.35) | 0.14 | 0.21 (0.13-0.35) | 0.14 | 0.21 (0.13-0.34) | 0.14 |
|  | Côte d'Ivoire | 0.22 (0.15-0.29) | 0.14 | 0.22 (0.13-0.33) | 0.15 | 0.24 (0.12-0.40) | 0.16 | 0.26 (0.12-0.46) | 0.17 | 0.27 (0.13-0.48) | 0.18 | 0.28 (0.13-0.52) | 0.19 |
|  | Djibouti | 0.01 (0.01-0.02) | 0.01 | 0.02 (0.01-0.02) | 0.01 | 0.01 (0.01-0.02) | 0.01 | 0.02 (0.02-0.04) | 0.02 | 0.09 (0.05-0.10) | 0.06 | 0.11 (0.11-0.11) | 0.07 |
|  | Equatorial Guinea | 0.35 (0.24-0.45) | 0.23 | 0.33 (0.21-0.46) | 0.22 | 0.30 (0.17-0.45) | 0.20 | 0.26 (0.15-0.40) | 0.17 | 0.24 (0.13-0.37) | 0.16 | 0.24 (0.10-0.41) | 0.16 |
|  | Gabon | 0.26 (0.12-0.47) | 0.17 | 0.22 (0.11-0.40) | 0.15 | 0.21 (0.11-0.36) | 0.14 | 0.20 (0.10-0.37) | 0.13 | 0.20 (0.10-0.37) | 0.13 | 0.21 (0.09-0.41) | 0.14 |
|  | Ghana | 0.26 (0.25-0.28) | 0.18 | 0.21 (0.19-0.24) | 0.14 | 0.17 (0.15-0.19) | 0.11 | 0.15 (0.13-0.17) | 0.10 | 0.14 (0.12-0.16) | 0.09 | 0.18 (0.14-0.21) | 0.12 |
|  | Guinea | 0.36 (0.27-0.46) | 0.24 | 0.34 (0.22-0.47) | 0.22 | 0.33 (0.21-0.49) | 0.22 | 0.33 (0.22-0.49) | 0.22 | 0.30 (0.19-0.46) | 0.20 | 0.29 (0.15-0.46) | 0.19 |
|  | Malawi | 0.18 (0.17-0.20) | 0.12 | 0.17 (0.15-0.18) | 0.11 | 0.16 (0.14-0.18) | 0.11 | 0.16 (0.13-0.18) | 0.10 | 0.16(0.10-0.21) | 0.10 | 0.18 (0.08-0.31) | 0.12 |
|  | Mali | 0.32 (0.30-0.35 | 0.22 | 0.23 (0.21-0.25) | 0.15 | 0.17 (0.15-0.19) | 0.11 | 0.14 (0.13-0.16) | 0.09 | 0.15 (0.12-0.18) | 0.10 | 0.21 (0.13-0.33) | 0.14 |
|  | Mozambique | 0.33 (0.31-0.35) | 0.22 | 0.32 (0.29-0.34) | 0.21 | 0.30 (0.27-0.33) | 0.20 | 0.28 (0.25-0.31) | 0.19 | 0.27 (0.22-0.32) | 0.18 | 0.29 (0.20-0.40) | 0.19 |
|  | Niger | 0.32 (0.19-0.49) | 0.21 | 0.31 (0.17-0.48) | 0.20 | 0.28 (0.16-0.44) | 0.18 | 0.26 (0.14-0.41) | 0.17 | 0.24 (0.14-0.42) | 0.16 | 0.24 (0.11-0.41) | 0.16 |
|  | Nigeria | 0.24 (0.22-0.27) | 0.16 | 0.24 (0.22-0.26) | 0.16 | 0.25 (0.23-0.26) | 0.16 | 0.25 (0.24-0.26) | 0.17 | 0.26 (0.22-0.29) | 0.17 | 0.28 (0.20-0.37) | 0.19 |
|  | Sierra Leone | 0.39 (0.36-0.43) | 0.26 | 0.39 (0.36-0.41) | 0.26 | 0.37 (0.32-0.42) | 0.25 | 0.37 (0.29-0.45) | 0.24 | 0.35 (0.22-0.48) | 0.23 | 0.33 (0.15-0.60) | 0.22 |
|  | ***Suppl Table cont.*** | **2015** | | **2016** | | **2017** | | **2018** | | **2019** | | **2020** | |
|  | **Country** | **PfPR 2-10y** | **PfPR 16-80y** | **PfPR 2-10y** | **PfPR 16-80y** | **PfPR 2-10y** | **PfPR 16-80y** | **PfPR 2-10y** | **PfPR 16-80y** | **PfPR 2-10y** | **PfPR 16-80y** | **PfPR 2-10y** | **PfPR 16-80y** |
|  | South Sudan | 0.23 (0.17-0.30) | 0.16 | 0.23 (0.13-0.31) | 0.15 | 0.25 (0.13-0.36) | 0.17 | 0.25 (0.12-0.38) | 0.17 | 0.29 (0.13-0.44) | 0.19 | 0.28 (0.12-0.43) | 0.18 |
|  | Togo | 0.35 (0.32-0.37) | 0.23 | 0.31 (0.28-0.33) | 0.20 | 0.25 (0.22-0.27) | 0.16 | 0.21 (0.18-0.25) | 0.14 | 0.19 (0.15-0.24) | 0.13 | 0.20 (0.13-0.29) | 0.13 |
|  | Uganda | 0.23 (0.22-0.24) | 0.15 | 0.22 (0.21-0.23) | 0.15 | 0.22 (0.20-0.23) | 0.14 | 0.19 (0.18-0.21) | 0.13 | 0.16 (0.14-0.18) | 0.11 | 0.20 (0.15-0.25) | 0.13 |
|  | Zambia | 0.17 (0.16-0.19) | 0.12 | 0.17 (0.14-0.19) | 0.11 | 0.15 (0.11-0.20) | 0.10 | 0.14 (0.10-0.20) | 0.10 | 0.14 (0.09-0.19) | 0.09 | 0.15 (0.10-0.22) | 0.10 |
| **Low** | Ethiopia | 0.03 (0.02-0.04) | 0.02 | 0.02 (0.01-0.03) | 0.01 | 0.02 (0.01-0.03) | 0.01 | 0.01 (0.01-0.02) | 0.01 | 0.01 (0.01-0.01) | 0.01 | 0.02 (0.01-0.03) | 0.01 |
|  | Gambia | 0.15 (0.10-0.22) | 0.10 | 0.08 (0.06-0.12) | 0.06 | 0.04 (0.03-0.05) | 0.03 | 0.04 (0.03-0.06) | 0.03 | 0.02 (0.02-0.03) | 0.02 | 0.04 (0.03-0.05) | 0.02 |
|  | Guinea-Bissau | 0.04 (0.02-0.10) | 0.03 | 0.04 (0.01-0.08) | 0.02 | 0.03 (0.01-0.08) | 0.02 | 0.03 (0.01-0.08) | 0.02 | 0.03 (0.01-0.08) | 0.02 | 0.05 (0.01-0.14) | 0.03 |
|  | Kenya | 0.05 (0.04-0.05) | 0.03 | 0.04 (0.04-0.05) | 0.03 | 0.04 (0.03-0.06) | 0.03 | 0.04 (0.03-0.06) | 0.03 | 0.04 (0.02-0.06) | 0.02 | 0.03 (0.02-0.05) | 0.02 |
|  | Madagascar | 0.06 (0.05-0.06) | 0.04 | 0.05 (0.05-0.06) | 0.03 | 0.05 (0.04-0.06) | 0.03 | 0.05 (0.04-0.06) | 0.03 | 0.05 (0.04-0.07) | 0.03 | 0.06 (0.04-0.09) | 0.04 |
|  | Mauritania | 0.07 (0.04-0.10) | 0.05 | 0.09 (0.06-0.13) | 0.06 | 0.06 (0.04-0.09) | 0.04 | 0.07 (0.04-0.10) | 0.04 | 0.05 (0.03-0.07) | 0.03 | 0.05 (0.04-0.08) | 0.03 |
|  | Rwanda | 0.03 (0.03-0.03) | 0.02 | 0.03 (0.03-0.04) | 0.02 | 0.04 (0.03-0.05) | 0.03 | 0.04 (0.03-0.05) | 0.03 | 0.03 (0.03-0.04) | 0.02 | 0.02 (0.02-0.03) | 0.02 |
|  | Senegal | 0.05 (0.03-0.06) | 0.03 | 0.03 (0.02-0.04) | 0.02 | 0.03 (0.03-0.04) | 0.02 | 0.04 (0.03-0.06) | 0.03 | 0.03 (0.02-0.05) | 0.02 | 0.04 (0.03-0.06) | 0.03 |
|  | Somalia | 0.02 (0.02-0.03) | 0.02 | 0.03 (0.03-0.04) | 0.02 | 0.05 (0.04-0.07) | 0.03 | 0.06 (0.05-0.09) | 0.04 | 0.07 (0.05-0.09) | 0.04 | 0.07 (0.05-0.10) | 0.04 |
|  | Sudan | 0.02 (0.02-0.03) | 0.02 | 0.02 (0.02-0.04) | 0.02 | 0.03 (0.02-0.06) | 0.02 | 0.03 (0.01-0.08) | 0.02 | 0.04 (0.02-0.08) | 0.02 | 0.04 (0.02-0.08) | 0.03 |
|  | Tanzania | 0.10 (0.09-0.10) | 0.07 | 0.09 (0.08-0.09) | 0.06 | 0.08 (0.07-0.09) | 0.05 | 0.07 (0.06-0.08) | 0.05 | 0.07 (0.05-0.09) | 0.05 | 0.08 (0.05-0.12) | 0.05 |
|  | Zimbabwe | 0.05 (0.04-0.06) | 0.03 | 0.03 (0.02-0.04) | 0.02 | 0.05 (0.03-0.06) | 0.03 | 0.02 (0.02-0.03) | 0.0158 | 0.03 (0.02-0.03) | 0.02 | 0.04 (0.03-0.05) | 0.03 |
| **Very low** | Botswana | 0.0001 | 0.0001 | 0.0003 | 0.0002 | 0.0009 | 0.0006 | 0.0003 | 0.0002 | 0.0001 | 0.0001 | 0.0004 | 0.0003 |
|  | Comoros | 0.0031 | 0.0020 | 0.0022 | 0.0014 | 0.01 (0.0-0.01) | 0.0038 | 0.02 (0.02-0.03) | 0.02 | 0.02 (0.02-0.03) | 0.02 | 0.01 (0.0-0.01) | 0.0041 |
|  | Eritrea | 0.0040 | 0.0027 | 0.01 (0.0-0.01) | 0.0036 | 0.01 (0.0-0.01) | 0.0037 | 0.01 (0.0-0.01) | 0.0033 | 0.01 (0.01-0.01) | 0.01 | 0.01 (0.01-0.01) | 0.01 |
|  | eSwatini | 0.0003 | 0.0002 | 0.0002 | 0.0002 | 0.0004 | 0.0003 | 0.0007 | 0.0004 | 0.0002 | 0.0001 | 0.0002 | 0.0002 |
|  | Namibia | 0.0037 | 0.0024 | 0.01(0.01-0-01) | 0.0052 | 0.02(0.02-0.03) | 0.02 | 0.01(0.01-0.02) | 0.01 | 0.01(0.0-0.02) | 0.0048 | 0.0047 | 0.0031 |
|  | South Africa | 0.0000 | 0.0000 | 0.0000 | 0.0000 | 0.0006 | 0.0004 | 0.0003 | 0.0002 | 0.0001 | 0.0001 | 0.0001 | 0.0001 |
